# Direct As(V) Determination Using Screen-Printed Electrodes Modified with Silver Nanoparticles

**DOI:** 10.3390/nano10071280

**Published:** 2020-06-30

**Authors:** Karina Torres-Rivero, Clara Pérez-Ràfols, Julio Bastos-Arrieta, Antonio Florido, Vicenç Martí, Núria Serrano

**Affiliations:** 1 Departament d’Enginyeria Química, Escola d’Enginyeria de Barcelona Est (EEBE), Universitat Politècnica de Catalunya, BarcelonaTEch (UPC), Av. Eduard Maristany 16, 08019 Barcelona, Spain; karina.torres.rivero@upc.edu (K.T.-R.); antonio.florido@upc.edu (A.F.); vicens.marti@upc.edu (V.M.); 2 Barcelona Research Center for Multiscale Science and Engineering, Av. Eduard Maristany 16, 08019 Barcelona, Spain; 3 Departament d’Enginyeria Química i Química Analítica, Facultat de Química, Universitat de Barcelona, Martí i Franquès 1-11, 08028 Barcelona, Spain; claraperezrafols@ub.edu; 4 Physical Chemistry, Technische Universität Dresden, Zellescher Weg 19, 01069 Dresden, Germany; 5 Fundació CTM Centre Tecnològic de Manresa, Plaça de la Ciència 2, 08243 Manresa, Spain; 6 Institut de Recerca de l’Aigua (IdRA), Universitat de Barcelona, Martí i Franquès 1-11, 08028 Barcelona, Spain

**Keywords:** arsenic determination, silver nanoparticles, anodic stripping voltammetry, screen-printed electrodes, water analysis

## Abstract

Carbon-nanofiber-based screen-printed electrodes modified with silver nanoparticles (Ag-NP-SPCNFEs) were tested in a pioneering manner for the direct determination of As(V) at low μg L^−1^ levels by means of differential pulse anodic stripping voltammetry. Screen-printed electrodes were modified with two different types of Ag-NPs, nanoseeds (NS), and nanoprisms (NPr) and characterized both microscopically and electrochemically. Furthermore, after optimizing the direct voltammetric determination of As(V), the analytical performance of considered sensors was compared for the direct determination of As(V). These results suggest that Ag-NS offer a better analytical response compared to Ag-NPr, with a detection and quantification limit of 0.6 and 1.9 µg L^−1^, respectively. The proposed methodology was validated using a spiked tap water sample with a very high reproducibility and good agreement with inductively coupled plasma-mass spectrometry (ICP-MS) measurements.

## 1. Introduction

Water pollution is an important problem that affects both developing and developed countries as a consequence of economic growth. In particular, contamination by metal ions causes significant environmental and health side effects, which are exacerbated by their high persistence, non- biodegradability, and ability to bioaccumulate [1]. Metal ions may enter the body through air, food, water, or skin absorption and, once in the body, they not only compete with but also displace essential minerals such as Zn, Mg, Ca, and Cu, interfering with organ system function [2,3]. Exposure to As can cause a variety of adverse health effects including hyperkeratosis, gastrointestinal symptoms, pulmonary disease, diabetes, cardiovascular problems, peripheral neuropathy, and cancer of the skin and internal organs [4]. Moreover, inorganic arsenic intake over a long period of time can lead to chronic arsenic poisoning (arsenicosis).

Arsenic, as a natural component of the earth’s crust, is widely allocated throughout the environment. Arsenic trace levels are present in rock, soil, seawater, etc., but high concentrations of arsenic can be found in mine drainage, coal fly ash, and smelter wastes [5]. More specifically, arsenic is a confirmed genotoxin, carcinogen, and globally the most significant chemical contaminant in drinking water [6]. Several arsenic species can be found in the environment, with four main different oxidation states (As^−3^, As^0^, As^+3^, and As^+5^). These present different hazard levels, as inorganic arsenic compounds are usually more toxic than organic arsenic compounds. In particular, soluble inorganic arsenic is highly toxic. Among inorganic forms, arsenic can mainly be found as two oxyanions: trivalent arsenite (H_3_AsO_3_) in the reducing environment and pentavalent arsenate (H_2_AsO_4_^−^) under oxidizing conditions. Arsenite compounds are reported to be more mobile, soluble, and toxic than arsenate [7,8,9].

Thus, people are exposed to elevated levels of inorganic arsenic through industrial processes, drinking contaminated water, eating contaminated food or food prepared or irrigated using contaminated water, and smoking tobacco. The World Health Organization (WHO) has fixed the guideline value of arsenic in drinking water at 10 µg L^−1^ [10].

However, considering that the concentration of arsenic species in real water samples is very low, sensitive detection methods are required to determine such levels of arsenic. Generally, these methods are based on atomic absorption spectrometry (AAS) [11], inductively coupled plasma-mass spectrometry (ICP-MS) [12], high-performance liquid chromatography with ICP-MS [13], and hydride generation atomic fluorescence spectrometry (HG-AFS) [14]. These techniques are mostly suitable for laboratory conditions but impractical for on-site analyses since they involve the use of complex instrumentation, have high costs, and are time-consuming [15]. In this sense, electrochemical techniques and particularly anodic stripping voltammetry (ASV) are more appropriate methodologies for the on-site determination of arsenic due to their low cost, fast analysis, and ease of incorporation in portable instrumentation. Furthermore, stripping techniques present low detection limits, high sensitivity, and can be implemented for arsenic speciation [16].

This last aspect is particularly important since, as mentioned before, different arsenic species present diverse toxicity. The direct ASV determination of As(III) is well-reported in the literature, whereas As(V) determination is usually more problematic due to its lower electrochemical response, leading to the quantification of As(V) by the difference between total arsenic and As(III) [17]. Consequently, direct ASV determination of As(V) is of great interest, especially if we take into account the potential and significant decrease of both the experimental time and the amount of reagents consumed (as no reducing agents are needed). Furthermore, unlike As(III) determination, As(V) measurements can be carried out without oxygen removal [17].

Most of the works dealing with the direct As(V) determination reported in the literature use gold-based electrodes in a highly acidic medium. Nevertheless, the cost of gold makes these electrodes highly expensive, which encourages the introduction of alternative metals with similar physical and chemical properties but more cost-effective. In this sense, silver is a material with great proven electrochemical features for metal ion determination [18]. In terms of silver-based electrodes, it is important to consider both the silver source (wire, film, ink, nanomaterials) and the support in which silver is contained. Regarding the substrate, nanotechnology has become a powerful tool to develop new sensing devices [19,20]. In particular, intrinsic characteristics of electrochemical sensors can be enhanced by modifying their surface with metal nanoparticles that confer better electrocatalytic properties compared with the non-modified sensors [21]. On the other hand, in terms of support, more classical bulk or film silver electrodes require tedious cleaning and polishing procedures to achieve good reproducibility. Accordingly, screen-printing technology presents some noticeable advantages such as its low-cost, disposable character, portability, and commercial availability [1].

Thus, in this work, a direct method for the voltammetric determination of As(V) is proposed based on the use of carbon-nanofiber-based screen-printed electrodes (SPCNFEs) modified by drop-casting with two different shaped silver nanoparticles (Ag-NPs) previously synthesized: nanoseeds (NS) and nanoprisms (NPr) [21]. The resulting modified sensors were microscopically and analytically characterized, and carbon-nanofiber-based screen-printed electrode modified with silver nanoseeds (Ag-NS-SPCNFE), as the optimal carbon-nanofiber-based screen-printed electrode modified with silver nanoparticles (Ag-NP-SPCNFE), was applied to the direct determination of As(V) ion in water samples by differential pulse anodic stripping voltammetry (DPASV).

## 2. Materials and Methods

### 2.1. Reagents

All the chemicals were of analytical reagent grade. Trisodium citrate, sodium polystyrene sulfonic acid (SPSS), and silver nitrate were supplied by Sigma-Aldrich (Munich, Germany), sodium borohydride from Panreac Applichem (Barcelona, Spain), and ascorbic acid from Scharlab (Barcelona, Spain). A total of 1 mg L^−1^ of As(V) solution was prepared by sequential dilution from a 1000 mg L^−1^ ICP standard supplied by Sigma-Aldrich (Munich, Germany). A total of 0.01 mol L^−1^ hydrochloric acid (pH 2.0) (Suprapur 30%, Merck, Munich, Germany) was used for pH control. All solutions were prepared with ultrapure water (18.2 MΩ cm) obtained from a Milli-Q plus 185 system Millipore (Millipore, Burlington, MA, USA).

Tap water samples were collected from the local water distribution network managed by Aigües de Barcelona Company (Barcelona, Spain; https://www.aiguesdebarcelona.cat/), and mostly using water coming from Llobregat and Ter Rivers.

### 2.2. Apparatus and Electrodes

DPASV measurements were performed in an Autolab PGSTAT204, attached to a Metrohm 663 VA Stand, or in a Multi Autolab/M204 Modular Multi Potentiostat/Galvanostat including an electrochemical impedance spectroscopy (EIS) unit, all from Metrohm (Herisau, Switzerland). The control of both setups as well as the required data treatment were performed by a personal computer with NOVA 2.1 software package (Metrohm, Herisau, Switzerland).

SPCNFEs with a diameter of 4 mm, purchased from Metrohm DropSens (ref. 110CNF, Llanera, Spain), were modified with Ag-NPs and used as working electrodes. Ag/AgCl/KCl 3 mol L^−1^ and a platinum wire were the reference and the counter electrodes, respectively, both supplied by Metrohm (Herisau, Switzerland). All voltammetric experiments were carried out in a glass cell without oxygen removal and at room temperature (22 ± 1 °C).

A Crison Basic 20 pH-meter (Hach Lange Spain, L’Hospitalet de Llobregat, Spain) was used for pH measurements.

Ag-NPs as well as the surface morphology of the SPCNFE electrodes were characterized using a JEM-2010 transmission electron microscope (TEM) from JEOL (Tokyo, Japan), and a Gemini scanning electron microscope (SEM) from ZEISS^®^ (Jena, Germany). TEM and SEM images were used to determine the size distribution of the obtained Ag-NPs, and the size distribution histograms were calculated by using the Image-J version 1.51m software by National Institutes of Health (NIH, Bethesda, MD, USA). An Agilent spectrophotometer model 8453 (Agilent Technologies, Waldbronn, Germany) was used to record the UV-VIS spectra of the Ag-NPs containing solutions. Inductively coupled plasma mass spectrometer model 7800 by Agilent Technologies (Santa Clara, CA, USA) was used for ICP-MS measurements.

### 2.3. Synthesis of Ag Nanoparticles

The preparation of Ag-NPs was performed in two phases, following a seed mediated methodology described in [22,23].

*Silver nanoseed (Ag-NS) preparation*: The Ag-NS were synthesized by mixing 5 mL of 2.5 mmol L^−1^ trisodium citrate, 0.25 mL of 500 mg L^−1^ SPSS, and 0.3 mL of 10 mmol L^−1^ aqueous sodium borohydride. Finally, a solution of 5 mmol L^−1^ silver nitrate was continuously added to the previous solution at a rate of 2 mL min^−1^ using a syringe pump from Kd Scientific, model KDS 510 (Holliston, MA, USA).

*Silver nanoprism (Ag-NPr) preparation*: The Ag-NPr were synthesized by adding 5 mL of Milli-Q water and 75 µL of 10 mmol L^−1^ ascorbic acid to either 800 or 1600 µL of the previous seed solution. Then, 3 mL of 0.5 mmol L^−1^ silver nitrate was continuously added to each aliquot at 1 mL min^−1^. To stabilize Ag-NPr solutions, 0.5 mL of 25 mmol L^−1^ sodium citrate was added.

### 2.4. Electrode Modification

The SPCNFEs were modified with Ag-NS or Ag-NPr using the drop-casting method described in [18,21]. Briefly, 40 µL of the solution of Ag-NS or Ag-NPr were dropped onto the working electrode surface and dried in an oven at 50 °C for 30 minutes. This modification approach was previously tested providing high repeatability (relative standard deviation, RSD, from 3.6% to 5.5% depending on the Ag-NPs considered) and reproducibility (RSD from 5.2% to 9.3% depending on the Ag-NPs considered) [18].

### 2.5. Electrochemical Measurements

Electrochemical impedance spectra were recorded in a solution containing 5 mmol L^−1^ K_3_[Fe(CN)_6_] and 0.1 mol L^−1^ KCl. The frequency range was set between 10 Hz and 1000 kHz with an alternating current (AC) amplitude of 10 mV.

The direct DPASV determination of As(V) was carried out in 0.01 mol L^−1^ of HCl (pH 2.0) by applying a deposition potential (E_d_) of −1.30 V (vs. Ag/AgCl) under stirring conditions during a deposition time (t_d_) of 120 s and scanning the potential from −1.3 to 0.0 V. A step potential of 5 mV, a pulse time of 50 ms, and a pulse amplitude of 50 mV were applied.

Linear calibration plots for As(V) determination were carried out by increasing metal ion concentrations in 0.01 mol L^−1^ HCl (pH 2.0).

Tap water samples were spiked with 20 μg L^−1^ of As(V). To perform the voltammetric determination of As(V), samples were acidified with 0.01 mol L^−1^ of HCl (pH 2.0), resulting in a final solution concentration of 10 μg L^−1^ of As(V). Four successive additions were made from a standard solution of 1 mg L^−1^ of As(V) and DPASV measurements were recorded under the above-mentioned electrochemical conditions.

### 2.6. Data Treatment

Peak areas were measured with NOVA 2.1 software, choosing a polynomial baseline that is generated by manually clicking on three points on the plot (initial point of the baseline, highest height, and final point of the baseline). Further calculations were made with EXCEL^®^.

## 3. Results and Discussion

### 3.1. Spectrophotometric and Microscopic Characterization

**Figure 1:**
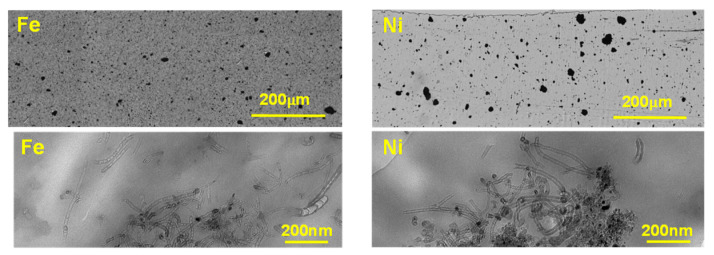
(**A**) UV–VIS spectra of Ag-NS, Ag-NPr (800 μL), and Ag-NPr (1600 μL); (**B**) TEM micrographs for Ag-NS and Ag-NPr (800 μL). SEM characterization and corresponding size distribution histograms for the Ag-NS (**C**,**D**) and Ag-NPr obtained using 800 μL (**E**,**F**) and 1600 μL (**G**,**H**) of Ag-NS as a precursor.

The Ag-NPs prepared by the seed mediated approach were first characterized by means of UV–VIS (Figure 1A) and transmission electron microscopy (TEM) (Figure 1B), which allowed the deduction of Ag-NPs shape and size. The different shapes and sizes observed for the three types of Ag-NPs are due to the first fast crystallization of nucleation seeds (Ag-NS) and different aliquots of Ag-NS being later used for further nucleation and growth in order to obtain larger nanocrystals (Ag-NPr) [24].

It can be seen in UV-VIS spectra that Ag-NS present a maximum absorption signal around 400 nm. Consequently, the more of these Ag-NS are used as a nucleation source for the Ag-NPr, the more similar the absorption spectra (see Figure 1A) and the larger the size expected (see size distribution histograms in Figure 1D,F,H). Here, the prepared nanoparticles present homogeneous size distribution as seen in TEM images (Figure 1B) and SEM micrographs (Figure 1C,E,G), with a calculated size of 11.23 ± 0.24 nm for Ag-NS. Regarding the size of the Ag-NPr, the values are 14.25 ± 0.28 and 16.46 ± 0.19 nm for Ag-NPr obtained using 800 and 1600 µL of Ag-NS as the precursor, respectively.

**Figure 2:**
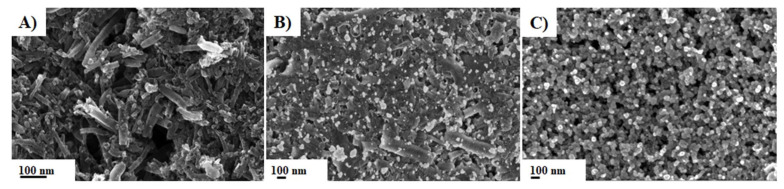
SEM micrographs for (**A**) Bare-carbon-nanofiber-based screen-printed electrodes (SPCNFEs) and electrodes modified by drop-casting: (**B**) Ag-NS-SPCNFE and (**C**) Ag-NPr-SPCNFE (800 µL).

The surface modification of SPCNFEs with Ag-NPs was assessed by scanning electron microscopy (SEM). Compared to the unmodified carbon nanofiber surface in the bare electrode (Figure 2A), Ag-NPs can be clearly spotted as white dots in both Ag-NS-SPCNFE (Figure 2B) and Ag-NPr-SPCNFE (800 µL) (Figure 2C). Furthermore, it can be observed that all Ag-NPs were homogeneously distributed all over the surface. Thus, the electrocatalytic enhancement of the screen-printed electrodes can be attributed to this spatial distribution of the different shaped Ag-NPs.

### 3.2. Electrochemical Characterization of Ag-NPs Modified SPCNFE

**Figure 3:**
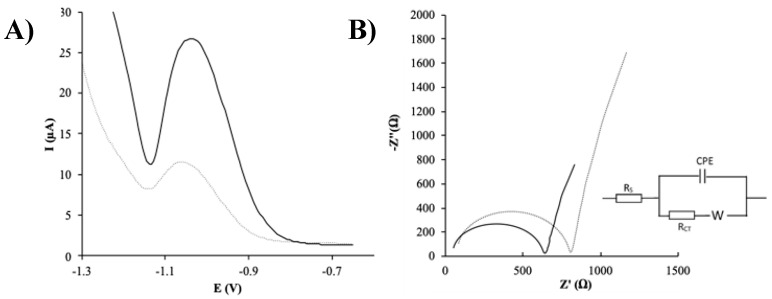
(**A**) Differential pulse anodic stripping voltammetric (DPASV) measurements of bare-SPCNFE and Ag-NS-SPCNFE sensors obtained for 50 and 25 µg L^−1^ of As(V) in 0.01 mol L^−1^ HCl pH 2, respectively. (**B**) Nyquist diagram for bare-SPCNFE and Ag-NS-SPCNFE in 5 mmol L^−1^ K_3_[Fe(CN)_6_] and 0.1 mol L^−1^ KCl with the corresponding equivalent circuit.

The effect of SPCNFE modification with Ag-NPs was initially evaluated by DPASV using Ag-NS as a model nanoparticle. For this purpose, the experimental conditions including E_d_ and t_d_ were first optimized in a 0.01 mol L^−1^ HCl solution containing 20 µg L^−1^ of As(V). The As(V) voltammetric peak increased as the E_d_ varied from −1.1 to −1.3 V and decreased for further negative E_d_ (results not shown). A t_d_ of 120 s was selected as a good compromise between peak area and analysis time. As can be seen in Figure 3A, at the optimized measuring conditions, a well-defined stripping peak could be observed for As(V) at ca. −1.0 V. Furthermore, the results shown in Figure 3A demonstrate that the modification of the electrode with Ag-NPs results in an important increase in the electrode response, which is crucial for the determination of the As(V) ion at low trace levels.

The modification of SPCNFE with Ag-NPs was also studied by EIS. Figure 3B shows the Nyquist plots obtained for both bare-SPCNFEs and Ag-NS-SPCNFEs, which were fitted to a Randles circuit. This is a basic equivalent circuit that considers the solution resistance (R_s_), the charge transfer resistance (RCT), the Warburg impedance (W), and a constant phase element (CPE), related to the non-idealities in the electrode surface [25]. The RCT values calculated from the semicircle diameter showed a significant decrease from 722 Ω in bare-SPCNFE to 589 Ω in Ag-NS-SPCNFE, indicating a higher electrocatalytic response and further demonstrating the effective attachment of Ag-NPs to the working electrode surface.

### 3.3. Analytical Performance of Ag-NPs Modified SPCNFE

The different synthesized silver nanoparticles (Ag-NS, Ag-NPr (800 µL), and Ag-NPr (1600 µL)) were used for the modification of the SPCNFE in order to determine which one gives better analytical response for As(V) quantification. Thus, Ag-NS-SPCNFE, Ag-NPr-SPCNFE (800 µL), and Ag-NPr-SPCNFE (1600 µL) were prepared to perform DPASV measurements.

**Figure 4:**
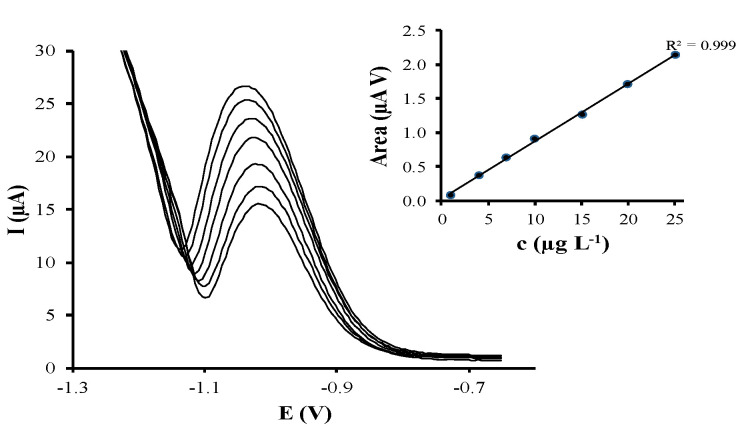
DPASV voltammograms of As(V) and its calibration plot (top-right inset) in 0.01 mol L^−1^ HCl pH 2 applying an E_d_ of −1.30 V and a t_d_ of 120 s using a Ag-NS-SPCNFE.

Calibration curves by DPASV were obtained by increasing the concentration of As(V) in a range from 1 to 25.1 µg L^−1^ and following the above optimized experimental conditions using a bare-SPCNFE and the different prepared Ag-NP-SPCNFE. Figure 4 shows, as an example, the evolution of DPASV signals of As(V) and its calibration plot (inset) using a Ag-NS-SPCNFE. Whereas a well-shaped and defined stripping peak close to −1.00 V that increases linearly with the As(V) concentration was obtained for all considered Ag-NP-SPCNFE sensors, the As(V) peak obtained using a bare-SPCNFE decreased when increasing the concentration, maybe due to the own oxidation of the working electrode, as it has no protective coating.

The limit of detection and quantification for DPV calibration curves performed using Ag-NS-SPCNFE, Ag-NPr-SPCNFE (800 µL), and Ag-NPr-SPCNFE (1600 µL) were calculated using the Miller and Miller methodology [26]. The results of the calibration parameters such as detection limits (LOD), linear ranges, sensitivities, and linearity are listed in Table 1. The limit of quantification (LOQ) is considered as the lowest value of the linear range.

As shown in Table 1, good linear responses of peak area vs. As(V) concentration were achieved using Ag-NS-SPCNFE, Ag-NPr-SPCNFE (800 µL), and Ag-NPr-SPCNFE (1600 µL). However, although LODs achieved for As(V) were at µg L^−1^ levels for all considered Ag-NP-SPCNFEs, the LOD value obtained using the Ag-NS-SPCNFE was much better than that provided by either Ag-NPr-SPCNFE (800 µL) or Ag-NPr-SPCNFE (1600 µL). Regarding previous works, the LODs obtained for the determination of As(V) using Ag-NS-SPCNFE are considerably lower in comparison to those reached by ASV using boron-doped diamond electrodes, 12 µg L^−1^ [27], and by sequential injection/ASV on gold-modified screen-printed carbon electrode, 2.3 µg L^−1^ [28]. However, it should be pointed out that no works are reported in the literature about the use of Ag-NP-based-sensors for As(V) determination. Moreover, Ag-NS-SPCNFE presents a much lower LOQ, leading consequently to a wider linear range compared to the other two tested sensors. Nevertheless, it should be pointed out that the highest value of the linear range is restricted to a lower concentration value (until 25.1 µg L^−1^) compared to those reached by Punrat et al. (until 100 µg L^−1^) [28] and by Nagaoka et al. (until 200 µg L^−1^) [27].

Regarding sensitivities (nA V µg^−1^ L), they were calculated as the slope value of the calibration curves of As(V) for the three considered Ag-NP-SPCNFEs, being Ag-NS-SPCNFE the most sensitive Ag-NP-based-sensor with a corresponding sensitivity value of 109 nA V µg^−1^ L.

From the reported calibration data, it can be concluded that all the considered Ag-NP-SPCNFEs could be fully suitable and a valuable option for the direct determination of As(V) at very low µg L^−1^ in environmental samples, with the addition of the particular characteristics of screen-printed electrodes, and particularly the three-electrode configuration that allows an easy connection to portable instrumentation making possible on-site analysis. However, Ag-NS-SPCNFE is the Ag-NP-based-sensor that exhibits the best analytical performance (lower LODs, wider linear ranges, and higher sensitivities), and therefore, it was selected as the optimal sensor for further determination of As(V) in water samples.

### 3.4. Application to the Analysis of a Real Sample: As(V) Spiked Tap Water

Ag-NS-SPCNFE suitability for the determination of As(V) in a real water sample was evaluated. The determination of As(V) ions was carried out in a spiked tap water sample by the standard addition calibration method. DPASV measurements using the above-mentioned conditions were performed, including four successive additions of the As(V) standard. It should be pointed out that DPASV measurements of non-spiked water samples did not present any As(V) signal.

**Figure 5:**
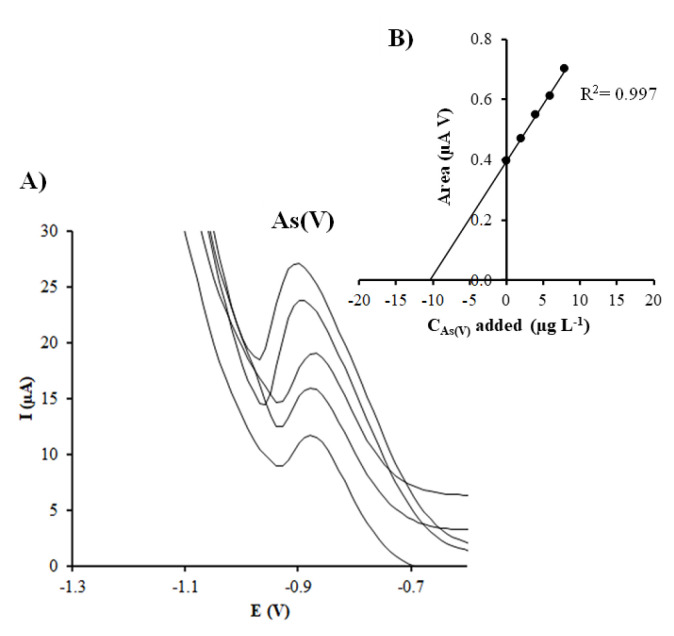
(**A**) DPASV measurements of As(V) in a spiked tap water sample on Ag-NS-SPCNFE at pH 2.0 applying an E_d_ of -1.30 V and a t_d_ of 120 s; and (**B**) As(V) standard addition plot.

Figure 5A shows representative voltammograms obtained for the analysis of the spiked tap water using Ag-NS-SPCNFE. A well-shaped As(V) peak was acquired and, as shown in the calibration curve (Figure 5B), a good correlation was achieved.

For the analysis of the spiked tap water sample using Ag-NS-SPCNFE, three replicates were considered. The result of the analysis was 10.04 µg L^−1^ (SD: 0.37 µg L^−1^). In order to test the accuracy of the proposed method, the spiked water sample was also analyzed by ICP-MS (10.7 µg L^−1^ (SD: 0.2 µg L^−1^)) and from a two-tailed *t*-test (null hypothesis: $\bar{X}$_ICP-MS_ = $\bar{X}$_DPASV_), it can be concluded that the results obtained from both techniques were statistically comparable with a confidence level of 99%.

These good results confirm the suitability of Ag-NS-SPCNFE for the direct determination of As(V) in water samples. Consequently, the use of Ag-NP-based sensors, and particularly Ag-NS-SPCNFE, is an excellent alternative to other sensors for the determination of As(V) at very low concentrations (µg L^−1^).

## 4. Conclusions

In this work, a DPASV method for the direct determination of trace As(V) based on the modification of SPCNFE with Ag-NPs has been proposed. Three different Ag-NPs with varying sizes and shapes were synthesized, microscopically characterized, and applied to the modification of SPCNFEs. Electrochemical characterization demonstrated that the modification with Ag-NPs significantly enhances the voltammetric response toward As(V).

The analytical performance of all three modified electrodes was compared, concluding that Ag-NS-SPCNFE, corresponding to spherical and smaller Ag-NPs, is the most suitable electrode to determine As(V) at µg L^−1^ levels given its lower LOD, higher sensitivity, and wider linear range. Regarding previous studies of As(V) direct determination, the LOD achieved in this investigation was much lower compared to other LODs previously reported. Moreover, Ag-NS-SPCNFE has the advantages of screen-printed electrodes, such as disposability, low cost, and the possibility to perform on-site analyses. Additionally, the direct determination of As(V) signifies a notable improvement in comparison to other proposed procedures in which As(V) is calculated as the difference between total As and As(III).

The feasibility of the DPASV method using Ag-NS-SPCNFE for As(V) determination was demonstrated using spiked water samples, achieving comparable results to those obtained by ICP-MS measurements with a good reproducibility deduced from the calculated standard deviation.

## Figures and Tables

**Table 1:** Calibration data for the determination of As(V) in hydrochloric acid pH 2.0 applying an E_d_ of −1.3 V and a t_d_ of 120 s.

	Ag-NS-SPCNFE	Ag-NPr-SPCNFE (1600 µL)	Ag-NPr-SPCNFE (800 µL)
**Sensitivity (nA V µg^−1^ L) ^a^**	109 (1)	93 (3)	29 (2)
**R^2^**	0.999	0.996	0.983
**Linear range (µg L^−1^) ^b^**	1.9–25.1	4.1–25.1	8.4–25.1
**LOD (µg L^−1^)**	0.6	1.2	2.5

^a^ The standard deviations are expressed in parentheses. ^b^ The lowest value of the linear range corresponds to the LOQ.

## References

[1] Barton J., García M.B.G., Santos D.H., Fanjul-Bolado P., Ribotti A., McCaul M., Diamond D., Magni P. (2016). Screen-printed electrodes for environmental monitoring of heavy metal ions: A review. Microchim. Acta.

[2] Järup L. (2003). Hazards of heavy metal contamination. Br. Med. Bull..

[3] Brown B., Ahsanullah M. (1971). Effect of heavy metals on mortality and growth. Mar. Pollut. Bull..

[4] Ahsan H. (2011). Arsenic in drinking-water. Kaohsiung J. Med. Sci..

[5] Goldberg S., Manning B.A., DeLaune R.D., Reddy K.R., Richardson C.J., Megonigal J.P. (2013). Speciation of arsenic(III)/arsenic(V) and selenium(IV)/selenium(VI) using coupled ion chromatography–hydride generation atomic absorption spectrometry. Methods in Biogeochemistry of Wetlands.

[6] Xiao L., Wildgoose G.G., Compton R.G. (2008). Sensitive electrochemical detection of arsenic (III) using gold nanoparticle modified carbon nanotubes via anodic stripping voltammetry. Anal. Chim. Acta.

[7] Prohaska T., Stingeder G., Cornelis R., Caruso J.A., Crews H., Heumann K.G. (2005). Handbook of Elemental Speciation II: Species in the Environment, Food, Medicine and Occupational Health.

[8] Hue N.V. (2013). Arsenic chemistry. Int. J. Phytoremediation.

[9] Komorowicz I., Barałkiewicz D. (2011). Arsenic and its speciation in water samples by high performance liquid chromatography inductively coupled plasma mass spectrometry—Last decade review. Talanta.

[10] World Health Organization (2011). Guidelines for Drinking-Water Quality.

[11] Tsalev D.L., Sperling M., Welz B. (2000). Flow-injection hydride generation atomic absorption spectrometric study of the automated on-line pre-reduction of arsenate, methylarsonate and dimethylarsinate and high-performance liquid chromatographic separation of their L-cysteine complexes. Talanta.

[12] Stetzenbach K.J., Amano M., Kreamer D.K., Hodge V.F. (1994). Testing the limits of ICP-MS: Determination of trace elements in ground water at the part-per-trillion level. Ground Water.

[13] Rubio R., Padró A., Albertí J., Rauret G. (1993). Determination of arsenic speciation by liquid chromatography-hydride generation inductively coupled plasma atomic emission spectrometry with on-line UV photooxidation. Anal. Chim. Acta.

[14] Shirkhanloo H., Mousavi H.Z., Rouhollahi A. (2011). Speciation and determination of trace amount of inorganic arsenic in water, environmental and biological samples. J. Chin. Chem. Soc..

[15] Kadara R.O., Tothill I.E. (2004). Stripping chronopotentiometric measurements of lead(II) and cadmium(II) in soils extracts and wastewaters using a bismuth film screen-printed electrode assembly. Anal. Bioanal. Chem..

[16] Liu Z.G., Huang X.J. (2014). Voltammetric determination of inorganic arsenic. TrAC Trends Anal. Chem..

[17] Zakharova E.A., Noskova G.N., Antonova S.G., Kabakaev A.S. (2014). Speciation of arsenic(III) and arsenic(V) by manganese-mediated stripping voltammetry at gold microelectrode ensemble in neutral and basic medium. Int. J. Environ. Anal. Chem..

[18] Pérez-Ràfols C., Bastos-Arrieta J., Serrano N., Díaz-Cruz J.M., Ariño C., de Pablo J., Esteban M. (2017). Ag nanoparticles drop-casting modification of screen-printed electrodes for the simultaneous voltammetric determination of Cu(II) and Pb(II). Sensors.

[19] Cinti S., Politi S., Moscone D., Palleschi G., Arduini F. (2014). Stripping analysis of As(III) by means of screen-printed electrodes modified with gold nanoparticles and carbon black nanocomposite. Electroanalysis.

[20] Chaudhuri R.G., Paria S. (2012). Core/shell nanoparticles: Classes, properties, synthesis mechanisms, characterization, and applications. Chem. Rev..

[21] Torres-Rivero K., Torralba-Cadena L., Espriu-Gascon A., Casas I., Bastos-Arrieta J., Florido A. (2019). Strategies for surface modification with Ag-shaped nanoparticles: Electrocatalytic enhancement of screen-printed electrodes for the detection of heavy metals. Sensors.

[22] Aherne D., Ledwith D.M., Gara M., Kelly J.M. (2008). Optical properties and growth aspects of silver nanoprisms produced by a highly reproducible and rapid synthesis at room temperature. Adv. Funct. Mater..

[23] Aherne D., Cara M., Kelly J.M., Gun’Ko Y.K. (2010). From Ag nanoprisms to triangular AuAg nanoboxes. Adv. Funct. Mater..

[24] Nikoobakht B., El-Sayed M.A. (2003). Preparation and growth mechanism of gold nanorods (NRs) using seed-mediated growth method. Chem. Mater..

[25] Serrano N., Prieto-Simón B., Cetó X., del Valle M. (2014). Array of peptide-modified electrodes for the simultaneous determination of Pb(II), Cd(II) and Zn(II). Talanta.

[26] Miller J., Miller J., Capella I. (2002). Métodos de Calibración en Análisis Instrumental: Regresión y Correlación.

[27] Nagaoka Y., Ivandini T.A., Yamada D., Fujita S., Yamanuki M., Einaga Y. (2010). Selective detection of As(V) with high sensitivity by as-deposited boron-doped diamond electrodes. Chem. Lett..

[28] Punrat E., Chuanuwatanakul S., Kaneta T., Motomizu S., Chailapakul O. (2013). Method development for the determination of arsenic by sequential injection/anodic stripping voltammetry using long-lasting gold-modified screen-printed carbon electrode. Talanta.

